# Acknowledgment of Reviewers 2024

**DOI:** 10.1089/whr.2024.32587.revack

**Published:** 2025-01-14

Critical evaluations of articles by expert reviewers make a vital contribution to ensuring the high quality of a journal’s content. The editorial leadership of *Women’s Health Reports* is very grateful for the support of the highly qualified peer reviewers who have dedicated their time and effort to reviewing our articles. We would like to show our appreciation by thanking the following individuals for their assistance with the review of articles for the journal in 2024.*

*Data through November 22, 2024.

Beau Abar

Abdulai Abubakari

Matthew Arnegard

Samantha Auerbach

Toluwalase Awoyemi

Montserrat  Ayala-Ramirez

Manal Badrasawi

Muttiah Barathan

Lior Baruch

Behnaz Basiri

Valleria Belleudi

Paola Bertuccio

Mehret Birru Talabi

Emma Blackson

Kimberly  Bloom-Feshbach

Allyson Bontempo

Frances Boyle

Brian Brost

Amanda Bruegl

Sarah Capelouto

Frances Casey

Giulia Casu

Bernard Chasekwa

R. Y. K. Cheung

Amy Copeland

Anita Cote

Stephanie Creasy

John Cyrus

Elysia Davis

Megan Donovan

Jonathan Douxfils

Robert Downs

Louis Dubeau

Chris Edwards

Jacqueline Ellison

Jennifer Erdrich

Jpsephine Exley

Derek Falk

Andre Farkouh

Jerome Federspiel

Megan Fockler

Jimi Francis

Edward Frongillo

Nimita Gaggar

Symielle Gaston

Christoph Gerlinger

Maria Julieta Germar

Mika Gisser

Linda Giudice

Dawn Goddard-Eckrich

Marji Gold

Augustin Goran

Barbara Gottlieb

Leo Han

Carmen Henning

Krystyna Holland

Jessie Hollingsworth

Xiaochu Hu

Marjorie Jenkins

Hendree Jones

Louise Kaplan

Nicole Karjane

Susan Kellogg-Spadt

Chi-Son Kim

Preben Kjölhede

Ram Kommagani

Abby Kruper

S. A. Käser

Suzanne Lea

Megan Lee

Raquel  Leirós-Rodríguez

Linda Lindström

Jeannette Littlemore

Adhemar  Longatto-Filho

Anne Loohuis

Angela Lupattelli

Shannon  MacLaughlan David

Constance Mao

Shinya Matsuzaki

Melissa Mauskar

Rose Maxwell

Sarmila Mazumder

Lisa McCorkel

Hayley McMahon

Michelle Meglin

Jane Meijlink

Stephanie Melkonian

Patricia Mota

Shillpa Naavaal

Anita Nelson

Rachael Nelson

Natalie Niemi

Dany Nkonlacl

Farhana Nosheen

Alice Ordean

Javier Ortiz

Joseph Osarfo

Joris Osinga

Jennifer Payne

Phoutdavone  Phimphasone-Brady

Meghan Pike

Greta Raglan

Nicklas Rasmussen

Kalyn Renbarger

Osvaldo Reyes

Cristina Rius

R. Paul Robertson

Robiyanto Robiyanto

Myra Robson

Elizabeth Rochin

Melissa Rosenstein

Mary Rysavy

Lea Sacca

Bethany Samuelson  Bannow

Jean Schoenen

Eleanor Schwarz

Lena Schönnesson

Yuri Sebastião

Priti Shah

Ishana Shetty

Robert Sidonio

Bhuchitra Singh

Katarzyna Skorupska

Brittany Smith

Silpa Srinivasulu

Dafna Sussman

Jonas Swartz

Thomas Amatey  Tagoe

Peter Takacs

Noriyuki Takahashi

Gebrehiwot Ayalew  Tiruneh

Doris Titus-Glover

Svetoslav Todorov

Vanessa Torbenson

Mai Linh Tran

Dani Tsevat

Elizabeth Unger

Jan-Paul  van Wingerden

Alicia VandeVusse

Carolyn Vandyken

Shannon Voogt

Thi Vu

Madhu Wagle

Heather Weinreich

Joanne Welsh

Sveinung  Wergeland Sørbye

Louise Wilkins-Haug

Alexei Wong

Hitoshi Yamauchi

Xuefen Yu

Sana Zeeshan

